# Practicalities of Using Non-Local or Non-Recent Multilocus Sequence Typing Data for Source Attribution in Space and Time of Human Campylobacteriosis

**DOI:** 10.1371/journal.pone.0055029

**Published:** 2013-02-06

**Authors:** Joost H. Smid, Lapo Mughini Gras, Albert G. de Boer, Nigel P. French, Arie H. Havelaar, Jaap A. Wagenaar, Wilfrid van Pelt

**Affiliations:** 1 Centre for Infectious Disease Control (CIb), National Institute for Public Health and the Environment (RIVM), Bilthoven, The Netherlands; 2 Institute for Risk Assessment Sciences, Utrecht University, Utrecht, The Netherlands; 3 Department of Veterinary Public Health and Food Safety, Istituto Superiore di Sanità, Rome, Italy; 4 Department of Veterinary Medical Sciences, Bologna University, Bologna, Italy; 5 Central Veterinary Institute of Wageningen UR, Lelystad, The Netherlands; 6 Massey University, Palmerston North, New Zealand; 7 Department of Infectious Diseases and Immunology, Utrecht University, Utrecht, The Netherlands; 8 World Health Organization Collaborating Centre for Reference and Research on Campylobacter/OIE Reference Laboratory for Campylobacteriosis, Lelystad/Utrecht, The Netherlands; The Australian National University, Australia

## Abstract

In this study, 1208 *Campylobacter jejuni* and *C. coli* isolates from humans and 400 isolates from chicken, collected in two separate periods over 12 years in The Netherlands, were typed using multilocus sequence typing (MLST). Statistical evidence was found for a shift of ST frequencies in human isolates over time. The human MLST data were also compared to published data from other countries to determine geographical variation. Because only MLST typed data from chicken, taken from the same time point and spatial location, were available in addition to the human data, MLST datasets for other *Campylobacter* reservoirs from selected countries were used. The selection was based on the degree of similarity of the human isolates between countries. The main aim of this study was to better understand the consequences of using non-local or non-recent MLST data for attributing domestically acquired human *Campylobacter* infections to specific sources of origin when applying the asymmetric island model for source attribution. In addition, a power-analysis was done to find the minimum number of source isolates needed to perform source attribution using an asymmetric island model. This study showed that using source data from other countries can have a significant biasing effect on the attribution results so it is important to carefully select data if the available local data lack in quality and/or quantity. Methods aimed at reducing this bias were proposed.

## Introduction

*Campylobacter* is the most common cause of bacterial gastroenteritis in the western world [1]. Several source attribution studies have been performed to quantify the relative contributions of different sources of infection to human campylobacteriosis. The results of these studies can be used for identifying those sources of infection that are the most promising targets for *Campylobacter*-reducing intervention efforts, as well as for measuring the impact of such efforts at varying levels of the transmission chain. Chicken has been indicated as the major contributor to the disease burden of human campylobacteriosis in most countries where source attribution studies pertaining to those geographical regions have been performed [2]–[5]. However, in other countries, ruminants have also been found to be important [6], [7]. As new *Campylobacter* sequence types (STs) emerge and the relative occurrence of the established ones change continually, attribution results may vary over time [8]. In addition, the human exposure to *Campylobacter* may vary as well, for example because of international travel and trade, changes in food consumption patterns and eating habits, either over space or time.

To estimate the proportion of human *Campylobacter* infections attributable to different sources, differences in the relative occurrence of bacterial subtypes in individual sources may be used. The *Campylobacter* spp. subtypes found in human cases and in food and environmental sources are compared to attribute human campylobacteriosis cases to sources. Multilocus Sequence Typing (MLST) [9] has been used as the typing method of choice in most recent studies [3]–[5], [7] as it displays a reasonable level of heterogeneity of *Campylobacter* STs among the different sources. Thus far, most published studies on *Campylobacter* source attribution have been performed in countries in which a relatively large number of local and recent *Campylobacter* spp. strains from animal and environmental sources have been isolated and typed with MLST. Yet, the set up of intensive sampling schemes and the examination of the collected samples to obtain *Campylobacter* MLST data from multiple sources is costly. As a result, *Campylobacter* MLST data and related source attribution studies are lacking in many countries. It has been noted that, although surprisingly robust [10], the use of non-recent or non-local data in attribution studies may introduce bias into the attribution results for human cases of campylobacteriosis within a country [11]. In addition, the use of a small-sized human or source dataset may result in uncertain and, therefore, less generalizable estimates.

In the Netherlands, *Campylobacter* MLST data have been collected from humans between 2002–2003 and 2010–2011 and from chickens between 2000–2007 and 2010–2011. In this study, we present these data and compare them with other published data from different countries. In addition, we analyze temporal changes in MLST frequencies of such data. Only a small number of local *Campylobacter* MLST reference strains were available from other sources than chicken. A method was proposed to select MLST datasets representing sources other than chicken from international studies to be used for source attribution purposes.

The aim of this study was to better understand the consequences of using non-local or non-recent MLST data for attributing domestically acquired human infections to their putative sources of origin. We investigated how the source attribution model used performs in absence of local or recent data, or when few data are available. Based on these analyses, we give recommendations about which and how many data from other countries should be used for obtaining reliable source attribution estimates if the available local data lack in quality and/or quantity.

## Materials and Methods

### Data

#### Campylobacter MLST data from the Netherlands

Data of laboratory-confirmed human cases of *Campylobacter jejuni* and *C. coli* infection in the Netherlands were obtained for two different periods. Between April 2002 and April 2003, stool samples were collected from 2858 *C. jejuni* and 257 *C. coli* human cases during a case-control study on risk factors for indigenous campylobacteriosis and salmonellosis, the so-called CaSa study [12]. Of these, 948 *C. jejuni* and 66 *C. coli* isolates were subsequently successfully typed with MLST [9] to be used for source attribution and source-specific risk characterization [2]. Of these, 743 cases (699 *C. jejuni* and 44 *C. coli*) were domestic cases, as the other cases had a recent history of foreign travel.

Isolates from more recent domestic human cases of campylobacteriosis routinely identified by the Dutch Regional Public Health Laboratories through passive surveillance were obtained between June 2010 and June 2011. In total, another 423 *C. jejuni* and 42 *C. coli* strains were typed using MLST.

In addition, 218 *Campylobacter* isolates from fresh retail chicken meat of Dutch origin, sampled between 2000 and 2007, were obtained. More recent chicken isolates of Dutch origin were obtained between 2010 and 2011. These consisted of 158 isolates from retail chicken meat and 24 isolates from layer hens, pooled together assuming that layers and chickens are a single reservoir (*Gallus gallus*). Isolates from other *Campylobacter* sources in the Netherlands were obtained between 2000 and 2006 (cattle, *n* = 9; pigs, *n* = 13; environmental water, *n* = 106). These isolates were also typed using MLST [9].

In this study, cases with a recent history of foreign travel were excluded and *C. jejuni*/*coli* data were given at the species level for humans but not for chicken isolates.

#### Campylobacter MLST data from international studies

A literature review was conducted to identify published studies that provide MLST data for *Campylobacter* isolates from human cases and from various sources in countries other than the Netherlands. It was required that the data in such studies were representative of the natural strain diversity and relative frequencies therein, in those countries. Therefore, studies presenting isolates which are subject to any form of selection (e.g. reporting of novel strains only) were excluded. Isolate collections used in this study are shown in Table 1.

**Table 1:** Number of isolates in published human (h) and source (s) datasets and (last column) bootstrapped similarities of the human data with the human data in the NL1 dataset.

Set	Reference^a^	Country (region)	Year	Human	Chicken	Cattle	Sheep	Pig	Environnent	PSI (95%CI)^b^ of human data to human data of NL1
NL1	[2]	Netherlands	h: 2002–2003; s: 2000–2007	**743**	**218**	**9**	0	**13**	**106**	
NL2	Data	Netherlands	2010–2011	**465**	**182**	0	0	0	0	0.41 (0.36–0.44)
SC	[4]	Scotland (Grampians)	2005–2006	278	239	**90**	**88**	**15**	**133**	0.40 (0.36–0.43)
CH2	[29]	Switzerland	2009	383	0	0	0	0	0	0.35 (0.31–0.38)
CH	[24]	Switzerland	h: 1993–2003; s: 2001–2002	76	77	**23**	0	**100**	0	0.31 (0.24–0.38)
CH3	[22]	Switzerland	2008	136	0	0	0	0	0	0.31 (0.27–0.35)
EN	[9]	England	h: 1977–2001; s: 1983–1999	355	73	**46**	**72**	**5**	**50**	0.30 (0.26–0.33)
NL3	[9]	Netherlands	h: 1996–1998; s: 1990–1999	76	53	3	0	0	0	0.29 (0.22–0.36)
NZ1	[3]	New Zealand (Manawatu)	2005–2008	502	331	99	140	0	104	0.26 (0.22–0.29)
SP	[6]	Spain	h: 2003–2009; s: 2003–2006	71	36	80	44	0	0	0.22 (0.15–0.27)
FI	[30]	Finland	h: 1996, 2002–2003; s: 2003	305	36	20	0	0	0	0.21 (0.18–0.25)
NZ2	[31]	New Zealand	2006	112	0	0	0	0	0	0.20 (0.15–0.26)
CUR	[26]	Curacao	1999–2000	205	0	0	0	0	0	0.19 (0.15–0.23)
AU	[27]	Australia (New South Wales)	1999–2001	153	0	0	0	0	0	0.18 (0.14–0.23)
US	[32]	USA (Michigan)	2003	17	13	0	0	0	0	0.14 (0.06–0.21)

a The datasets are ordered in decreasing similarity of the human isolates with the NL1 human data. Numbers of isolates that are written in bold were used in the baseline source attribution analysis.

b Proportional Similarity Index, with 95% confidence intervals.

### Comparison of Datasets

#### Analysis of diversity

The distributions of ST frequencies in different datasets were compared visually by stacking the frequency bars of the most common STs found in the different studies next to each other. In addition, the frequency distributions of the most common STs and clonal complexes (CCs) in different datasets were compared with one another to allow for genetically close relationships between STs within the same CC to be evidenced. Approximate confidence intervals (CIs) for the ST or CC frequencies were calculated using bootstrapping [13].

The proportional similarity index (PSI, or Czekanowski index) [14] was used to measure the similarity of frequencies of STs between the different datasets. The PSI is expressed as PSI = 1 − 0.5 ∑*_k_* |*P_k_* − *Q_k_*|, where |*P_k_* − *Q_k_*| is the absolute value of the difference in the relative frequency of MLST genotype *k* in dataset *P* compared to its frequency in dataset *Q*. The values of PSI range from 0 to 1, with 0 indicating that both distributions have no types in common and 1 that both distributions are completely equal. CIs for the PSI were also calculated using bootstrapping [13].

#### Principal component analysis

In addition to the numerical similarities measured by the PSI between datasets, a principal component analysis (PCA) [15] provides additional insights towards which STs are the main contributors to the differences observed between the different datasets. Briefly, the original coordinate system, in which each axis represents the relative frequency of one ST in the datasets, is linearly transformed by PCA. In the transformed coordinate system, most variability is explained by the first coordinate (the first principal component), the second largest variability is explained by the second coordinate, etc. The proportion of the variability that is explained by the *n*th coordinate equals the fraction of the *n*th eigenvalue out of the summed total of all eigenvalues of the transformation matrix. A plot of the transformed axes shows which STs are most relevant for explaining the differences between the datasets. If the first dimensions of the transformed system explain the majority of variability, only these need to be plotted.

### Source Attribution

#### Asymmetric island model

The large effective population size of *Campylobacter* causes frequent mutation despite a relatively low mutation rate per allele [16]. Also, *Campylobacter* recombines [17] and migrates from one host to another [18]. With the Asymmetric Island (AI) model [5], the parameters describing these genetic changes within, and drift between, the source populations are inferred using Bayesian inversion. Subsequently, they are used for comparing one group of isolates (the attributable population) to other groups (the source populations). For each case, the AI model estimates a relative assignment posterior probability (*Pr*) to originate from each source. The proportion of human infections attributed to a given source is calculated as the average *Pr* over all cases. The AI model has been used for source attribution in a number of published studies [5], [19], [20] and has been reported to provide results with a relatively high level of confidence [19].

#### Baseline attribution analysis

In the baseline attribution analysis, the attributable population consisted of the 1208 non-travel related Dutch human cases in 2002–2003 and 2010–2011. The source populations were defined by the available MLST source data from the Netherlands supplemented with MLST source data from a selection of other published studies. Supplementary source data were used from countries where the human isolates were most similar to Dutch human isolates, as indicated by the PSI. Isolates that were used in the baseline attribution analysis are printed in bold in Table 1. The augmented dataset is composed in such a way that there were 168 isolates for cattle, 160 for sheep, 133 for pig and 289 for the environment. Chicken data from countries other than the Netherlands were not used because sufficient Dutch data were available for this source.

#### Advanced attribution analyses

Typically, the available *Campylobacter* MLST data for source attribution are imperfect [2]. Source data are in fact scarcer than human data in many countries because these are not collected routinely. To verify the impact of imperfect source data, the following scenarios were tested:

• Source attribution with non-local source data
• Source attribution with limited source data

The impact of using non-local source data was assessed by performing the following source attribution analyses of Dutch human cases and comparing their results to the baseline attribution results. First, chicken data from countries relatively close to the Netherlands (UK, here Scotland and England, and Switzerland) were used instead of the domestic chicken data. The effect of using chicken data from countries further away from the Netherlands (New Zealand, Finland and the US) was also investigated. Ultimately, domestic chicken and chicken from Scotland, England and Switzerland were considered as distinct sources in the attribution analysis.

The impact of using non-local source data was further studied by letting non-local chicken isolates be the attributable population, and attributing these using the source populations as defined in the baseline attribution analyses (bold numbers in Table 1). This type of analysis is called self-attribution, and can also be used to test the statistical power of the attribution model [19]. In this case, a high similarity between the non-local and local chicken isolates and a high statistical power of the model should result in a self-attributed proportion of the chicken isolates that is close to 1.

Self-attribution was also used to study the impact of using source data with a limited sample size. Of the 400 chicken isolates used in the baseline attribution, 250 were randomly selected to be the attributable population. Experiments indicated that the effect of modifying this initial split of the chicken data on the attribution results was negligible; thus, only one random split was considered in the following experiments. The remaining 150 chicken isolates and 150 randomly selected isolates from the remaining source populations (as defined in the baseline attribution model) were the reduced source populations. Subsequently, self-attribution was carried out. Self-attribution of the same 250 chicken isolates was also done with random subsets of 100, 75 and 50 isolates from each source population to explore the effects of using smaller-sized source datasets. To account for variability in the attribution results caused by the random subset selection, self-attribution was done 10 times for every subset of the source population, while keeping the attributable population of 250 chicken isolates constant.

Finally, a source attribution analysis based on the minimum possible non-recent and non-local data was performed. This was made by letting the human cases in 2002–2003 be the attributable population and using the corresponding Dutch source data (NL1 dataset in Table 1) supplemented with only the most similar non-Dutch source data (SC dataset in Table 1).

## Results

### Temporal Variation

**Figure 1:**
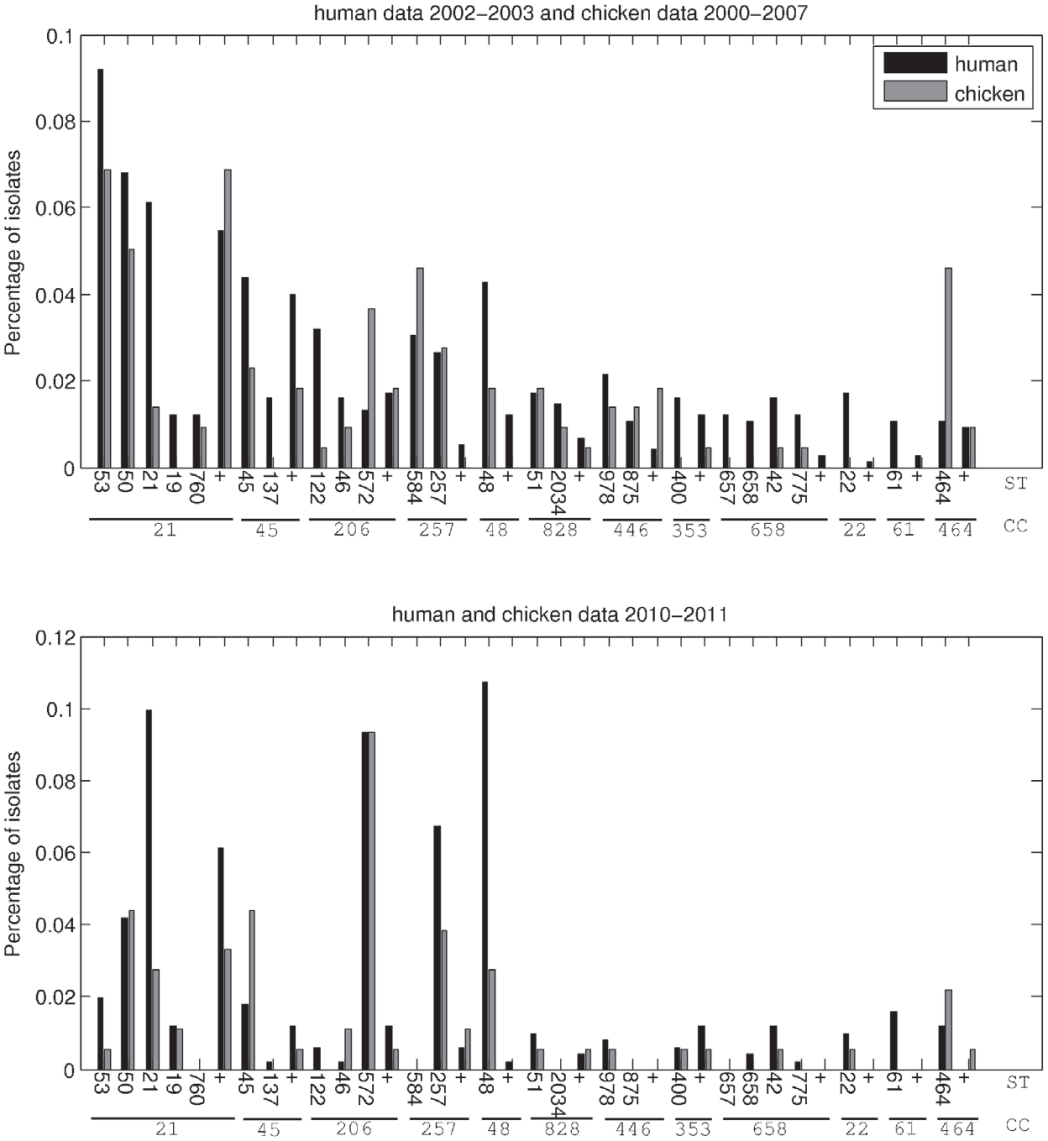
Most common STs in human and chicken isolates in The Netherlands in two time periods. Only the contributions of those CCs including STs that were found in the human data of 2002–2003 in proportions over 0.01 are represented. The contributions of less frequent STs within these CCs are summed and presented by the “+” symbol; the contributions of other CCs are omitted. For the human data of 2002–2003 the presented CCs make up for 83% of all isolates. For the chicken data of 2000–2007 and the human and chicken data of 2010–2011, these CCs make up for 65%, 67% and 52% of all data, respectively.

A large variety of STs was found in the Dutch human and chicken data. In Figure 1, the contributions of those CCs including STs that were found in the human data of 2002–2003 in proportions over 1% (accounting for 83% of all isolates) are represented together with the contributions of the same STs within these CCs for chicken data of 2000–2007 and for human and chicken data of 2010–2011; these CCs accounted for 65%, 67% and 52% of all isolates, respectively.

Among the 743 human isolates from 2002–2003, 161 different STs were observed. The five most frequent STs were ST53 (9.2%), ST50 (6.8%), ST21 (6.1%), ST45 (4.4%) and ST48 (4.3%). Among the 218 chicken isolates from 2000–2007, 87 different STs were found, the five most common STs being ST2483 (7.8%), ST53 (6.9%), ST50 (5.0%), ST584 (4.6%) and ST464 (4.6%). In the 465 human isolates from 2010–2011, 129 different STs were observed. The five most frequent STs were ST48 (10.7%), ST21 (9.9%), ST572 (9.3%), ST257 (6.7%) and ST50 (4.2%). Among the 182 chicken isolates from 2010–2011, 82 different STs were found, the five most common STs being ST2274 (11.5%), ST572 (9.3%), ST50 (4.4%), ST45 and ST257 (3.8%).

**Figure 2:**
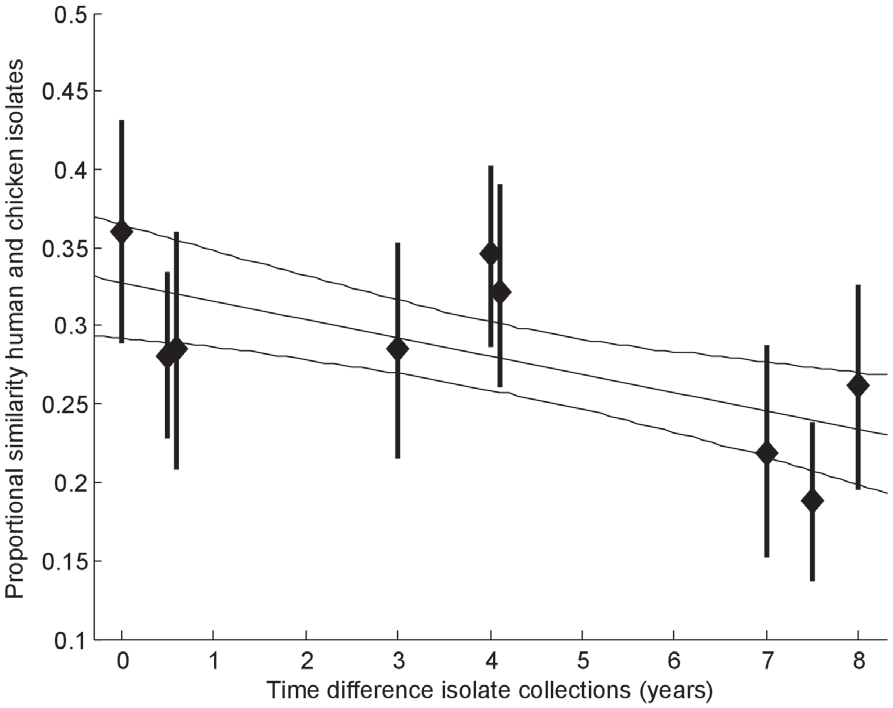
Similarity of STs in chicken and in human isolates from samples collected in different years. The *x*-axis gives the absolute difference between years in which the isolates from human cases and chicken were obtained. To enhance the size of the sample subsets, chicken isolates collected between 2000 and 2004 were aggregated and assigned to be collected in 2002, those collected between 2005 and 2007 were assigned to be collected in 2006, and those collected in 2010–2011 were assigned to be collected in 2010. Human isolates were arranged in three groups: 2002, 2003, and 2010–2011. The *y*-axis represents the PSI between those isolates collections.

The PSI was used as a tool to quantify the (dis)similarity between recent and non-recent isolates. Figure 2 indicates that the STs isolated from chicken and human cases are increasingly dissimilar as the period between which the samples were taken increases. The linear decrease is borderline significant with a mean slope of −0.011 (95% CI: −0.018 to −0.003). PSI was also calculated for chicken data between 2000–2004 and 2005–2007 (PSI = 0.24, 95% CI: 0.11–0.38), between 2000–2004 and 2010–2011 (0.20, 0.07–0.33), and between 2005–2007 and 2010–2011 (0.34, 0.22–0.46). Although the 95% CIs overlap one another, a trend is notable towards dissimilarity of chicken data as the period between which the samples were taken increases.

### Geographical Variation

**Figure 3:**
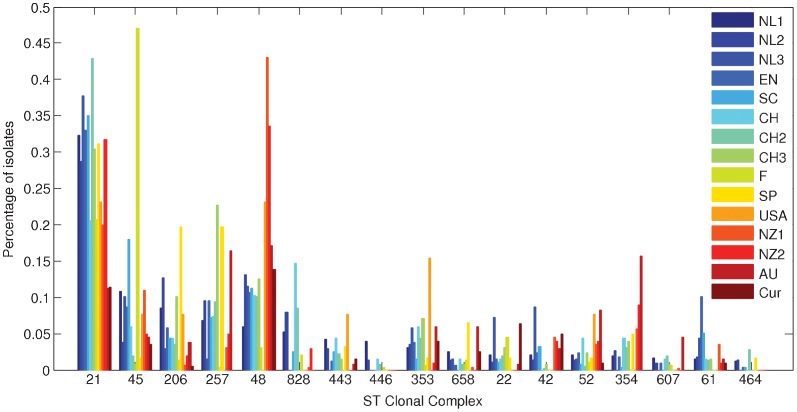
Bar chart of frequency distributions of the most prevalent CCs in 12 human datasets. Only CCs of which a prevalence higher than 10% was found are plotted.

The frequency distributions of the most common CCs in human datasets published in the international literature are shown in Figure 3. The most commonly found CC in England is CC21, followed by CC45, CC48 and CC257. For the Dutch human data, these were also important CCs in 2002–2003 as well as in 2010–2011. CC21 was less common in human cases in other countries, in particular in Australia and in the US. CC48 was remarkably prominent in New Zealand. This is mainly due to CC48 member ST474, which accounted for 30% and 29% of all human cases in the two New Zealand studies, respectively. ST45, the founder strain of CC45 was by far the most common ST in Finland, accounting for 28% of the human cases. CC354 member ST528, which is frequently reported in New South Wales, Australia, was not reported in the other studies.

The analysis of similarity of the Dutch human data from 2002–2003 with the human data from other datasets shows that they are most similar to Dutch human data from 2010–2011, followed by human data from Scotland, England and Switzerland (Table 1). In general, the Dutch human data were less similar to data from Finland, Spain and the considered non-European countries. This was expected because of the differences in geographical distance. All international datasets were significantly different from the Dutch human data from 2002–2003, as can be seen by the fact that the similarity 95% CI within these Dutch data does not overlap any other similarity confidence interval (Table 1).

**Figure 4:**
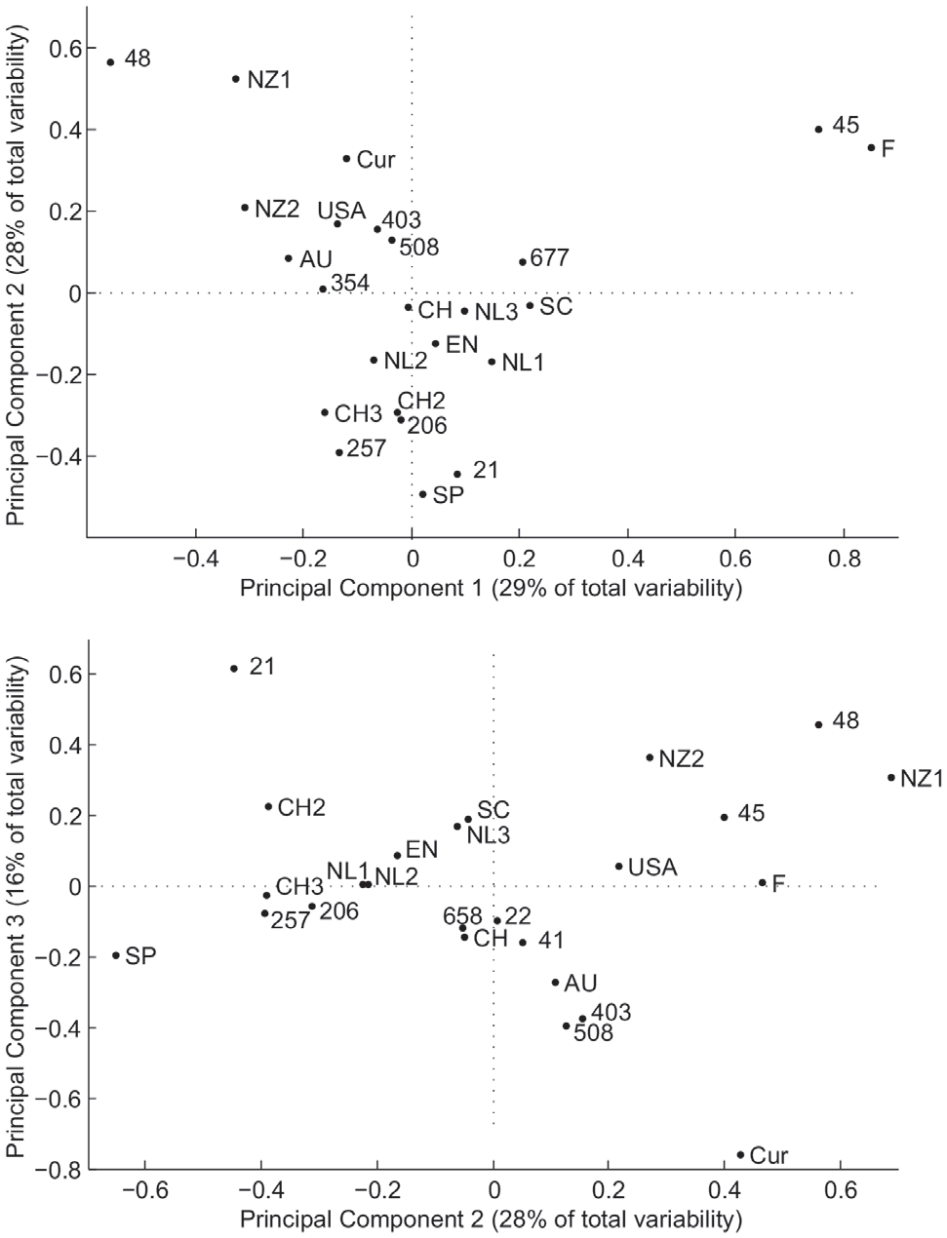
PCA transformed vectors of CC frequencies in 12 human datasets. The first, second and third PCA transformed dimensions explain together 73% of the total variability in the data. Weighted sums of the CC frequency distributions of the human isolates in the different datasets reported in Table 1 are plotted in the first two (upper graph) and in the second two (lower graph) dimensions of the PCA transformed space.

In Figure 4, the first three dimensions of the PCA transformed system of ST frequency vectors are plotted. ST474 sets the datasets from New Zealand apart from other datasets and ST21 sets datasets from Switzerland slightly apart from other datasets. The Finnish dataset is set apart from other datasets by a high prevalence of ST45 and the dataset from Curacao is set somewhat apart from other datasets due to a high prevalence of ST508. Evaluation of the eigenvalues of the transformation matrix obtained in the PCA showed that the first three dimensions of the transformed coordinate system explain about 73% of the variability of ST frequencies between the datasets.

### Attribution Analyses

**Figure 5:**
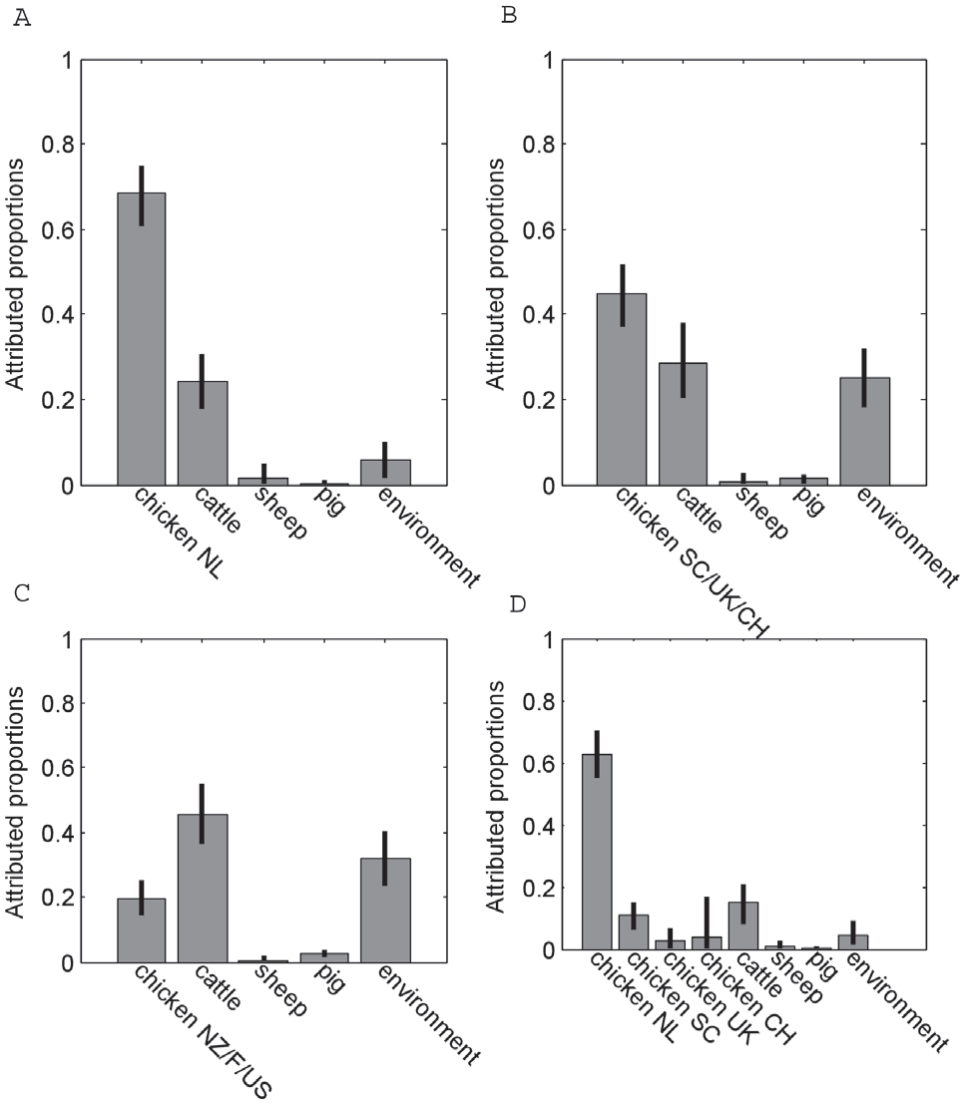
Overall mean probability (%) and 95% confidence interval for human *C. jejuni* and *C. coli* infections to originate from chicken, cattle, pig, sheep, and the environment. A. Baseline attribution results (see main text); B. Attribution results with Dutch chicken isolates replaced by chicken isolates from Scotland, the UK and Switzerland; C. Attribution results with Dutch chicken isolates replaced by chicken isolates from New Zealand, Finland and USA; D. Attribution results with Dutch, Scottish, English and Swiss chicken isolates as separate *Campylobacter* reservoirs.

In the baseline attribution analysis (Figure 5A), of all 1208 human cases of campylobacteriosis, 68% (95% CI: 61–74%) was attributed to chicken, 24% (18–31%) to cattle, and 6% (2–10%) to the environment, while the contributions of sheep and pig were only minor (2% together). If the Dutch chicken data were replaced by chicken data from Scotland, England and Switzerland (Figure 5B), then the importance of chicken for human disease decreased to 45% (37–52%), whereas the contributions of non-chicken sources increased. Replacement of the Dutch chicken data by chicken data from New Zealand, Finland and the US (Figure 5C) greatly reduced the inferred role of chicken for human disease (20%, 14–25%), leading to cattle being the most important source (45%, 36–55%), followed by the environment (32%, 23–40%). When data from domestic chicken and data from Scottish, English and Swiss chicken were considered as separate sources in the model (Figure 5D), then it is evident that there is much more overlap of MLST genotypes between the domestic chicken and Dutch human isolates (63%, 55–70%) rather than non-Dutch chicken (17% together).

If the self-attribution analysis were done with domestic chicken as the attributable population and the source populations the same as in the baseline attribution analysis, then 89% (81–95%) of these isolates were attributed to the right source. If chicken isolates from Scotland, England and Switzerland were assigned as the attributable population, then the percentage of correct self attribution was 62% (47–75%). Similarly, if chicken isolates from New Zealand, Finland and the US were assigned as the attributable population then 62% (49–73%) of these isolates were attributed to the right source.

**Figure 6:**
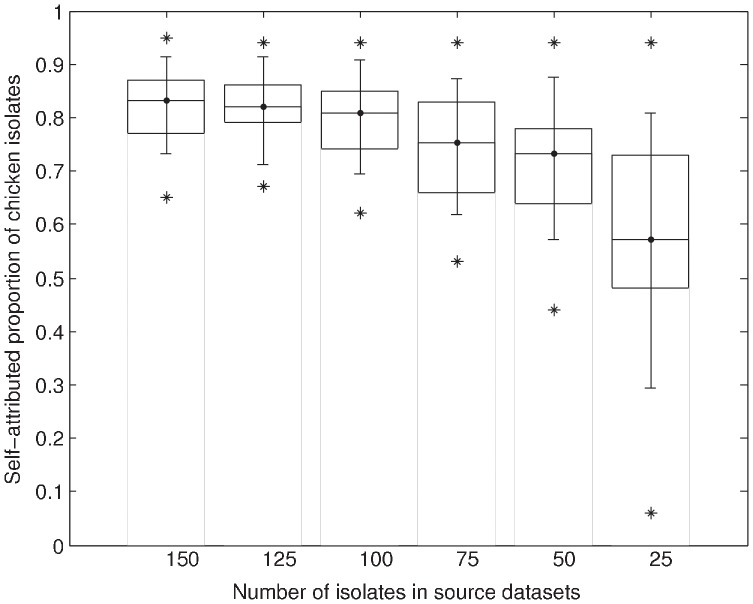
Statistics of the self-attributed proportions of 250 chicken isolates for reduced source datasets of size *n* (on *x*-axis). Every reduced dataset is generated from the original dataset by randomly removing isolates from an original set of 150. The boxes indicate variability in the mean attributed proportions over the 10 different reduced datasets per model and per reduction factor. Indicated are the minimal, maximal and average means. The whiskers indicate the average 2.5% and 97.5% confidence limits over the different reduced datasets. The star-symbols represent the minimum 2.5% limit and the maximum 97.5% limit.

Figure 6 shows the impact of using limited source data. It is seen that the variability over the mean attributed proportions (caused by randomly generating reduced datasets) increases for smaller subsets of the original source data. This is evident as the random effects increase for these smaller subsets. Also the statistical power of the AI model decreases if fewer data are available, which leads to a larger uncertainty. This implies that the confidence of the attribution results decreases as fewer data are available. The statistical power of the attribution model was fairly robust for smaller-sized source datasets until a minimum number of 100 isolates per source. If fewer than 100 isolates are available per source then the statistical power of the attribution model decreased substantially. For an average 2.5% confidence over 50% of correct source attribution, it is advisable that more than 25 isolates per source are used.

In the attribution analysis based on the minimum possible non-recent and non-local data (where the word “minimum” here refers to the supplementary non-Dutch source data used in the model and not to the sample size), 63% (95% CI: 56–69%) of the 743 human cases of 2002–2003 was attributed to chicken, 25% (19–32%) to cattle, and 11% (6–15%) to the environment, while the contributions of sheep and pig were again minimal (1% together).

## Discussion

We presented the results of a study in which *Campylobacter* isolates from Dutch human patients (*n* = 1208) and Dutch chicken (*n* = 400) collected between 2002–2003 and 2010–2011 were typed using MLST. The large size of this dataset provided the opportunity to perform a multitude of analyses aimed at defining the effect of time and geographical location on the diversity of the *Campylobacter* population. Other reservoirs for *Campylobacter* were less well sampled in the Netherlands. Therefore, non-local source data were used to supplement the Dutch ones in order to attribute the human infections to the different sources. A practical method was also proposed to select such supplementary data with the aim of minimizing potential biases of the attribution estimates. This method is based on the assumption that if the human data between different countries and time periods resemble one another (as revealed by PSI and PCA), then also will their respective source data, which may therefore be borrowed interchangeably for the purposes of source attribution. Inherent to this way of choosing the source data is the assumption that the consumption patterns and exposure pathways from sources to humans are similar in the Netherlands and in the countries/time periods from which the supplementary source data were collected, and that diversity between the human datasets can only be explained by intrinsic differences in the genotype distribution and by sampling uncertainty.

The attribution analyses showed that chicken was the most important source of human campylobacteriosis in the Netherlands, accounting for 61–74% of the human cases in the baseline model where the two human datasets for 2002–2003 and 2010–2011 were pooled based on their high similarity and the fact that the corresponding source data covered on average the whole time period. This is in line with findings from previous source attribution studies conducted in several other countries [2], [3], [7], [21], [22]. Nevertheless, our analyses suggest that the high proportion of human cases attributed to chicken and the smaller proportions of cases attributed to non-chicken sources (which are less intensively sampled in the Netherlands) may depend on the origin of the source data included in the model. When domestic chicken data were replaced by chicken data from countries showing the closest possible human MLST profiles to those of the Netherlands, i.e. Scotland, England and Switzerland, the ranking of sources remained the same as that of the baseline model but the contribution of chicken to human cases decreased considerably. This was more evident and the ranking of sources was even reversed when domestic chicken data were replaced by chicken data from countries with human MLST data less similar to those of the Netherlands, i.e. New Zealand, Finland and the US. Moreover, when Dutch, Scottish, English and Swiss chicken data were included as separate sources, it became apparent that domestic chicken is much more important than foreign chicken in accounting for domestic human cases. Together these findings suggest that the further in region and time one takes the source data, the more their MLST profiles will differ, and the smaller will be the estimated proportions of human cases attributable to those sources that were sampled less close in time and space to the human cases.

ST50 is shared as a common ST among the human and chicken isolates collected in the periods 2002–2003 and 2010–2011, and results from the AI model showed that human cases with ST50 had a 90% probability of having been infected by chicken or by strains with chicken origin. This ST belongs to CC21, which is reported to have a relatively wide distribution across many host species but slightly more dominant in ruminants [23]. Other STs belonging to this complex are ST21 and ST53. ST21 was more common in human cases than in chicken in both periods. Results of the AI model showed that human cases with ST21 were slightly more likely to have been infected by ruminants (*Pr* = 0.51) than by chicken (*Pr* = 0.43). The decline of ST53 in samples from chicken, being the most frequent ST in samples from 2000–2007 but a minor ST in samples from 2010–2011, coincided with a decline of this ST in the human samples as well. A similar decline was seen for ST584 in the chicken and in the human samples. This may indicate the importance of chicken as the source for campylobacteriosis caused by these STs. Results of the AI model confirmed that the probability that these STs originated from chicken was 0.84 and 0.97 for ST53 and ST 584, respectively. In contrast, ST2274 was increasingly common in chicken samples, which coincides with an increase of this ST in the human samples. Results from the AI model showed that human cases with ST2274 were most likely to have been infected by chicken (*Pr* = 0.97). The predominant STs in human data in the Netherlands in 2002–2003 were ST53 and ST50, both belonging to CC21. Also in other studies [7], [20], [21], [24], these strains were reported to be common in human patients.

By comparing the human datasets from several countries to the Dutch human data, it was concluded that the importance of the differences in ST frequencies is correlated with the geographical distance between the countries, with the data from nearby European countries being generally more similar than data from more distant countries with respect to the Netherlands, such as New Zealand, Australia and the US. PCA was proposed as a method to show in a visually appealing way the difference in occurrence of STs in different studies. The transformed vector representing the Dutch human data is relatively close to the origins of these PCA plots. This indicates that the 2002–2003 Dutch human dataset does not contain one or more CCs in markedly different frequencies than the average frequency distribution over all datasets that were considered. This may be caused by the ease of traveling and trade within the European Union, which leads to a larger exposure to *Campylobacter* from reservoirs present in European countries. However, limited exposure to this international diversity of *Campylobacter* strains may occur in people living in countries where there is a less open national market such as New Zealand or Australia, or where less international importation of meat products, including poultry meat, takes place, such as Spain or Finland. Indeed, approximately 8% and 11% of the total amount of meats available for consumption in 2000–2009 in Spain and Finland were imported, respectively, and these figures are considerably lower than those for the Netherlands (∼45%), the UK (∼30%), and Switzerland (∼16%) [25]. Human isolates from Curacao were taken from Guillain-Barré cases [26], which is a particular subset of campylobacteriosis cases. These may be reasons that studies in these countries show different frequencies of certain CCs compared to the averaged frequencies over all studies, which may be seen by the larger distance from the origins in the PCA plots. Also the CCs that set the studies from these countries apart from other studies are shown in the PCA plots. Indeed, CC48, in particular the CC48 member ST474, is reported in New Zealand more frequently than in other countries [20], ST528, belonging to CC354, is more frequently reported in New South Wales, Australia [27], and the CC45 member ST45 is more frequently reported in Finland [7].

The PCA shows only those CCs which explain the largest variation between the different datasets. Yet, in many studies the same STs (e.g. ST21, ST22, ST48 and ST 257) turn up as the predominant strains. This provides evidence to the suggestion made by Mickan et al [27] that some STs have a global distribution, while others are restricted in their distribution to a more local environment, however the “local STs” may be more associated with countries with less international travel and trade [28].

The results of our study show that it is recommended to have over 100 isolates per food source to perform source attribution using the AI model in order to have satisfactory statistical power. More detailed research questions with respect to attribution estimates might ask for more precision, hence a larger strain set. If this amount of data is not available for each potential source when using only recent and domestic data, then the investigator may be forced to use non-recent or non-local data. We have shown that the MLST data supply for *Campylobacter* within a food source is subject to dynamic changes in time and over geographical location; thus, in principle, this introduces temporal and geographical bias into the study.

As the AI model is based on a population genetics approach, source data collated from studies that show large variations between isolates obtained from the same sources but from different datasets may distort the gene frequencies upon which source attribution relies [5]. Sample size may impact on such variation by letting certain sources to exhibit relatively more unique (with respect to humans and the other sources) genotypes than others; thus, more intense sampling of small-sized sources is generally desirable, as oversampling certain sources relative to the others does not seem to affect the point estimates but only their accuracy [5]. Indeed, the source dataset becomes denser and better defined in terms of representative genotypes by increasing the number of samples. Therefore, notwithstanding the distortion of gene frequencies due to the pooling of source datasets from different studies, this may become less important with increasing sample size.

In conclusion, we have shown that, even on a small time-scale, MLST data within two sources become increasingly dissimilar as the time between different datasets are collected increases so that the AI model may underestimate the importance of a source whose data are not collected contemporaneously with the human cases to be attributed. Temporal bias can be minimized by choosing the most recent data that are available for a source. In addition, the AI model may underestimate the importance of sources from which non-local source data were used. A coarse rule is that this bias increases with the geographical distance between the countries in which the attribution is performed and from which source data are used. Nevertheless, our results show that geographical distance is not the only factor, and it may act together with factors related to travel and trade between countries. It also has been found that association of genotypes to a particular host is reported to be stronger than their association to a geographical location [10]. Our results show that, although this may make the consequences of geographically biased data less severe, it does not fully compensate for them (Figure 5). In general, the extent to which this bias is a matter of concern depends on how detailed (in time and region) is the research question to be addressed. A method based on the comparison of human isolates from different studies using PSI and PCA was proposed to select non-recent and non-local MLST datasets for the purposes of source attribution while minimizing potential biases.
